# Supplementary material for: The effect of intrapartum prolonged oxygen exposure on fetal metabolic status: secondary analysis from a randomized controlled trial
Source: Front Endocrinol (Lausanne). 2023 Jun 27;14:1204956. doi: 10.3389/fendo.2023.1204956 (PMC10335765; doi:10.3389/fendo.2023.1204956)
Supplement: Supplementary file 3 [file DataSheet_3.zip › Result-X101SC21092977-Z01-J001-B1-42/2.MetAnnotation/KEGG/meta_neg.KEGG.Anno.pdf]

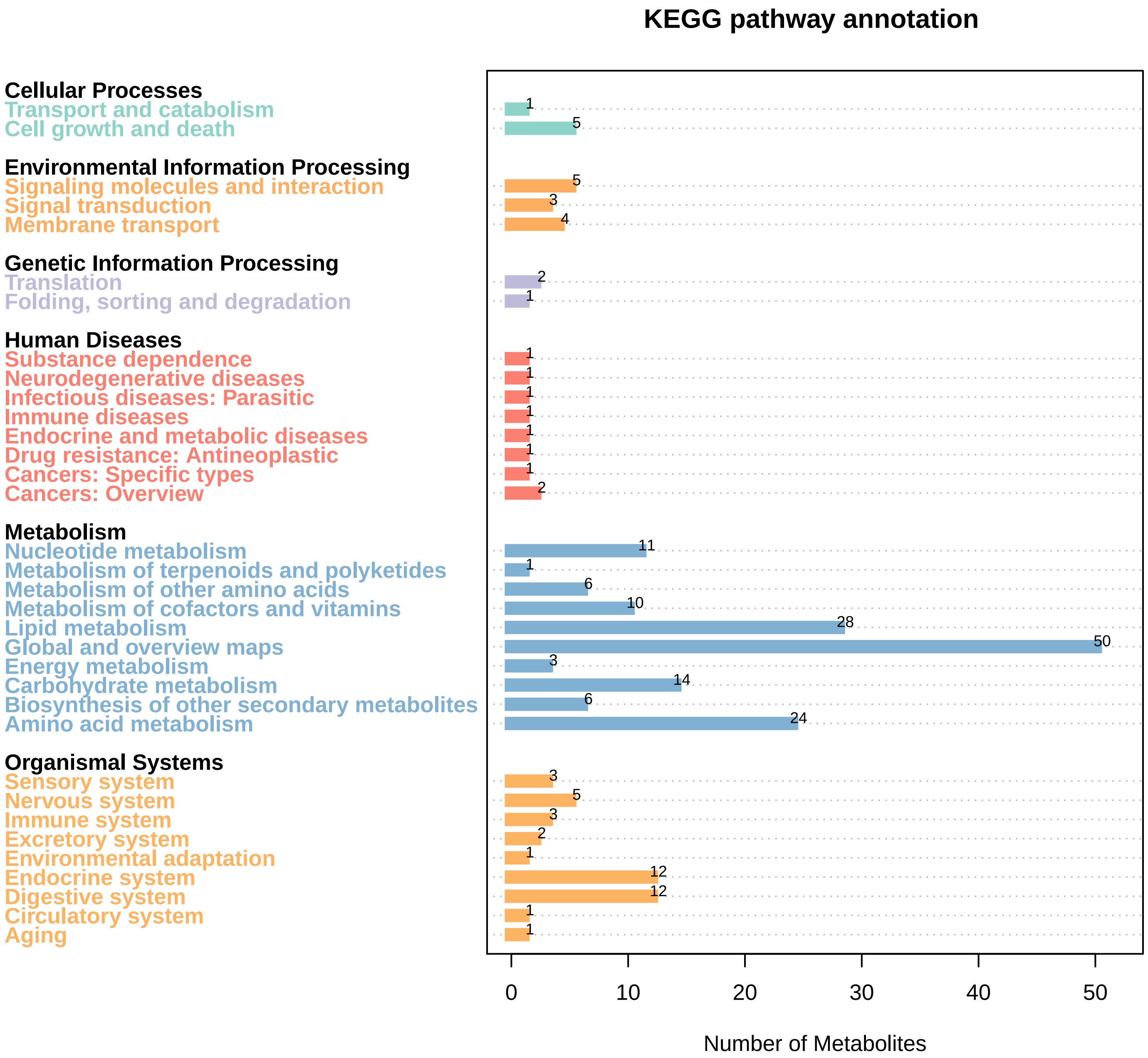
KEGG pathway annotation
Cellular Processes
Transport and catabolism
Cell growth and death
Environmental Information Processing
Signaling molecules and interaction
Signal transduction
Membrane transport
Genetic Information Processing
Translation
Folding, sorting and degradation
Human Diseases
Substance dependence
Neurodegenerative diseases
Infectious diseases: Parasitic
Immune diseases
Endocrine and metabolic diseases
Drug resistance: Antineoplastic
Cancers: Specific types
Cancers: Overview
Metabolism
Nucleotide metabolism
Metabolism of terpenoids and polyketides
Metabolism of other amino acids
Metabolism of cofactors and vitamins
Lipid metabolism
Global and overview maps
Energy metabolism
Carbohydrate metabolism
Biosynthesis of other secondary metabolites
Amino acid metabolism
Organismal Systems
Sensory system
Nervous system
Immune system
Excretory system
Environmental adaptation
Endocrine system
Digestive system
Circulatory system
Aging
1
5
5
3
4
2
1
1
1
1
1
1
1
1
2
11
1
6
10
28
50
3
14
6
24
3
5
3
2
1
12
12
1
1
0
10
20
30
40
50
Number of Metabolites
